# Prevalence, Enterotoxigenic Potential and Antimicrobial Resistance of *Staphylococcus aureus* and Methicillin-Resistant *Staphylococcus aureus* (MRSA) Isolated from Algerian Ready to Eat Foods

**DOI:** 10.3390/toxins13120835

**Published:** 2021-11-24

**Authors:** Omar Amine Mekhloufi, Daniele Chieffi, Abdelhamid Hammoudi, Sid Ahmed Bensefia, Francesca Fanelli, Vincenzina Fusco

**Affiliations:** 1 Laboratory of Hygiene and Animal Pathology, Institute of Veterinary Sciences, University of Tiaret, Tiaret 14000, Algeria; amineomarvet@gmail.com (O.A.M.); hammoudiabdelhamid@yahoo.fr (A.H.); 2 National Research Council of Italy, Institute of Sciences of Food Production (CNR-ISPA), 70126 Bari, Italy; francesca.fanelli@ispa.cnr.it (F.F.); vincenzina.fusco@ispa.cnr.it (V.F.); 3 Food, Water and Environmental Bacteriology Laboratory, Pasteur Institute of Algeria, Algiers 16000, Algeria; sbensefia@hotmail.fr

**Keywords:** *Staphylococcus aureus*, staphylococcal enterotoxin, enterotoxin gene cluster (*egc*), staphylococcal food poisoning, staphylococcal chromosomal cassette *mec* (SCC*mec*), methicillin resistant *Staphylococcus aureus* (MRSA), antibiotic resistance, toxic shock syndrome toxin-1, 16S-23S rDNA intergenic spacer region PCR (ISR-PCR), ready-to-eat foods

## Abstract

*Staphylococcus aureus* causes a foodborne intoxication due to the production of enterotoxins and shows antimicrobial resistance, as in the case of methicillin-resistant strains (MRSA). Herein, we analyzed 207 ready-to-eat foods collected in Algeria, reporting a *S. aureus* prevalence of 23.2% (48/207) and respective loads of coagulase positive staphylococci (CPS) ranging from 1.00 ± 0.5 to 5.11 ± 0.24 Log CFU/g. The 48 *S. aureus* isolates were widely characterized by staphylococcal enterotoxin gene (SEg)-typing and 16S-23S rDNA intergenic spacer region (ISR)-PCR, as well as by detecting *tst* and *mec*A genes, genetic determinants of toxic shock syndrome toxin-1 and methicillin resistance, respectively. We found that the *S. aureus* isolates belonged to seven different SEg-types harboring the following combinations of genes: (1) *sel*W, *sel*X; (2) *egc* (*se*G, *se*I, *se*M, *se*N, *se*O), *sel*W, *sel*X; (3) *se*A, *se*H, *se*K, *se*Q, *sel*W, *sel*X; (4) *se*B, *sel*W, *sel*X; (5) *se*D, *sel*J, *se*R, *sel*W, *sel*X; (6) *se*H, *sel*W, *sel*X, *sel*Y; and (7) *se*A, *egc*, *sel*W, *sel*X, while among these, 2.1% and 4.2% were *tst-* and *mec*A- (staphylococcal chromosomal cassette *mec*-type IV) positive, respectively. Selected strains belonging to the 12 detected ISR-types were resistant towards antimicrobials including benzylpenicillin, ofloxacin, erythromycin, lincomycin, tetracyclin, kanamycin, oxacillin, and cefoxitin; 8.3% (1/12) were confirmed as MRSA and 16.7% (2/12) were multidrug resistant. The present study shows the heterogeneity of the *S. aureus* population in Algerian ready-to-eat foods as for their toxigenic potential and antimicrobial resistance, shedding the light on the quality and safety related to the consume of ready-to-eat foods in Algeria.

## 1. Introduction

Food-associated health problems, caused by food contamination or malnutrition, have a worldwide impact on public health and economy [1]. Particularly, foodborne diseases (FBDs) represent a major issue for public health, and there is a growing number of outbreaks and sporadic cases of disease associated with various type of foods [2,3,4]. Furthermore, FBDs have a significant economic impact on both the industry and the public health system in terms of loss of productivity, cost of treatment, and food safety governance [5]. On top of that, FBDs are responsible for nearly 600 million illnesses and 420,000 deaths each year [6].

*Staphylococcus aureus* is one of the major pathogens of humans. It causes various diseases including staphylococcal food poisoning (SFP), toxic shock syndrome and other systemic diseases, bacteremia, pneumonia, and skin and soft tissue infections, to cite a few [7,8]. Its pathogenicity is promoted via several virulence factors, such as staphylococcal enterotoxins (SEs), toxic shock syndrome toxin-1, haemolysins, and fibronectin-binding proteins [9]. This microorganism is not only considered as the most pathogenic of staphylococci but, as reported by Havelaar et al. [10] and Fusco et al. [11], it is also acknowledged among the top 10 causes of bacterial FBDs, therefore playing a major role in terms of food safety [12].

Staphylococcal enterotoxins (SEs), produced by enterotoxigenic *S. aureus* strains, are a super family of pyrogenic exotoxins that share structural and functional similarities, causing vomiting, diarrhea, and cramps upon ingestion. To date, at least 28 SEs and staphylococcal enterotoxin-like toxins (SEls) have been identified [13]. SE and SEls are globular, single polypeptides with molecular weights ranging from 22 to 29 kDa. They can be encoded in prophages, plasmids, or chromosomal pathogenicity islands. The location of the *se* and *sel* genes on mobile genetic elements presents an additional risk factor in *S. aureus* food intoxication, due to possible horizontal gene transfer [14].

Foods that have been frequently incriminated in SFP include meat and meat products, poultry and egg products, milk and dairy products, salads, bakery products, particularly cream-filled pastries and cakes, and sandwich fillings [15]. However, according to le Loir et al. [16], foods implicated with SFP vary from country to country, particularly due to variation in consumption and food habits. In particular, food contamination with *S. aureus* and its SEs is mainly due to its ability to enter the food chain through contaminated raw material, inappropriate handling of processed food, and failure to maintain the cold chain [17].

Moreover, *S. aureus* is a well-known bacterium that develops antibiotic resistance [18] due to its ability to acquire a variety of resistance mechanisms towards antimicrobial agents [19], such as the resistance to methicillin. The latter mainly depends on the acquisition of the staphylococcal chromosomal cassette *mec* (SCC*mec*) harboring the *mec*A gene that encodes for penicillin-binding protein 2a (PBP2a) [20] that has low affinity for β-lactam antibiotics. In recent years, methicillin-resistant *S. aureus* (MRSA) strains have been recovered from several animal-derived foods such as poultry, pork, and beef meats, suggesting that foods may serve as a reservoir and source of MRSA [21]. Apart from direct transmission between humans and animals, the latter being considered natural reservoirs of this organism, transmission of MRSA might occur via exposure to, or ingestion of, contaminated foods [22,23]. Therefore, *S. aureus* and MRSA are considered a significant public health concern given their ability to contaminate foods and to colonize and infect both humans and animals [23,24].

Various ready-to-eat products are becoming increasingly popular in developing countries, particularly in the metropolitan areas. The prevalence and characteristics of enterotoxigenic *S. aureus* in ready-to-eat food products has been studied in different parts of the world [25,26,27]; however, as highlighted by Lozano et al. [28], studies on foodborne *S. aureus* in Africa are limited and, to date, mainly restricted to few countries, such as South Africa, which in general is reported to contribute to the most part of the African investigations, followed by Egypt and Nigeria [29]. Moreover, the available studies are focused primarily on the assessment of the prevalence of *S. aureus* and corresponding loads in various foods [28], while a further characterization is not systematically addressed. Thus, the information on enterotoxigenic *S. aureus* and MRSA from food in the African continent is, to date, fragmented and still incomplete. In order to overcome these limits, the present study aims to investigate the prevalence, the enterotoxigenic potential, and the antimicrobial resistance of *S. aureus* in ready-to-eat foods sampled in Algeria, Africa.

## 2. Results

### 2.1. Prevalance of Staphylococcus aureus in Algerian Ready to Eat Foods

In this study, *S. aureus* was found in 23.2% (48/207) of ready-to-eat foods sampled in Algeria. High prevalence was found in meat/fish-based foods (38.2%, 21/55), followed by vegetable-based foods (22.2%, 16/72), cereals (17.6%, 3/17), pastries (16.3%, 7/43), and various foods (milk- and egg-based foods) (5.0%, 1/20) (Table 1).

The coagulase positive staphylococci (CPS) counts in the *S. aureus* positive samples showed a median of 3.38 ± 0.94 Log CFU (colony forming unit)/g, ranging between 1.00 ± 0.5 and 5.11 ± 0.24 Log CFU/g as minimum and maximum counts, respectively. Pastries showed the highest CPS counts (between 2.84 ± 0.11 and 5.11 ± 0.24 Log CFU/g; median: 3.57 ± 0.80 Log CFU/g), while various foods (milk- and egg-based foods) showed the lowest (1.70 ± 0.10 Log CFU/g) (Table 1).

### 2.2. 16S-23S rDNA Intergenic Spacer Region (ISR)-PCR, Staphylococcal Enterotoxin Gene (SEg)-Typing and Presence of tst and mecA Genes

The 48 *S. aureus* isolated from the ready-to-eat foods sampled in Algeria are listed in Table 2.

**Figure 1:**
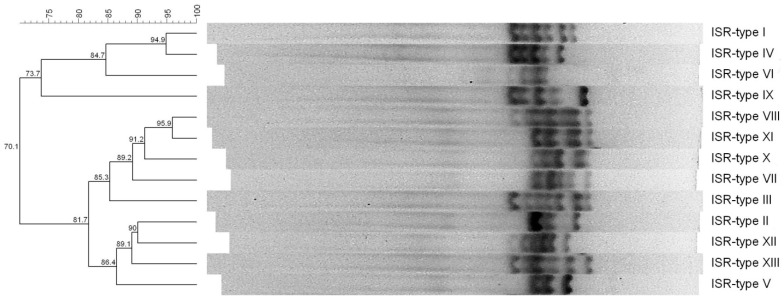
UPGMA (unweighted pair group method with arithmetic mean) dendrogram of the different ISR-types of the *S. aureus* isolated from Algerian ready-to-eat foods (ISR-types I to XII), compared to *S. aureus* type strain DSM 20231 (ISR-type XIII).

The 16S-23S rDNA intergenic spacer region (ISR)-PCR allowed us to identify 12 different ISR-types, as shown in Figure 1.

Thirty-nine point six percent of the *S. aureus* (19/48) belonged to ISR-type I; 12.5% (6/48) to ISR-type III; 10.4% (5/48) to ISR-type IV and VII, respectively; 8.3% (4/48) to ISR-type IX; and 4.2% (2/48) to ISR-type II and X, respectively, while 2.1%, of the *S. aureus* isolates belonged to ISR-types V, VI, VIII, XI, and XII, which were represented by one isolate each (1/48, respectively) (Table 2).

The staphylococcal enterotoxin gene (SEg)-typing allowed us to detect seven different SEg-types (Table 2). The most prevalent was SEg-type 1 (*sel*W, *sel*X) detected in 52.1% (25/48) of the *S. aureus* that belonged to three different ISR-types (I, IV, and VI). It was followed by SEg-type 2 [*egc* (*se*G, *se*I, *se*M, *se*N, *se*O), *sel*W, *sel*X] (16.7%) (8/48) observed in *S. aureus* belonging to ISR-types II, V, IX, and XII, SEg-type 3 (*se*A, *se*H, *se*K, *se*Q, *sel*W, *sel*X) (12.5%) (6/48) in *S. aureus* belonging to ISR-types III, SEg-type 4 (*se*B, *sel*W, *sel*X) (10.4%) (5/48) in *S. aureus* belonging to ISR-type VII, and SEg-type 6 (*se*H, *sel*W, *sel*X, *sel*Y) (4.2%) (2/48) in *S. aureus* belonging to ISR-type X. The least prevalent were SEg-types 5 (*se*D, *sel*J, *se*R, *sel*W, *sel*X) and 7 [*se*A, *egc* (*se*G, *se*I, *se*M, *se*N, *se*O), *sel*W, *sel*X] (2.1%) (1/48), which were detected in *S. aureus* belonging to ISR-type VIII and XI, respectively (Table 2).

One out of the 48 *S. aureus* isolates (2.1%) that belonged to ISR-type XII was positive for the *tst* gene, and two *S. aureus* (4.2%) belonging to ISR-type X were positive for the *mec*A gene (Table 2).

It is noteworthy that *S. aureus* isolates belonging to the same ISR-type harbored the same genes as detected by PCRs (Table 2).

In regard to the prevalence of the *se* and *sel* genes, *sel*W and *sel*X were detected in all the isolates (48/48), followed by the enterotoxin gene cluster (*egc*) and its related genes (*se*G, *se*I, *se*M, *se*N, *se*O) (18.8%, 9/48), *se*H (16.7%, 8/48), *se*A (14.6%, 7/48), *se*K and *se*Q (12.5%, 6/48 respectively), *se*B (10.4%, 5/48), *sel*Y (4.2%, 2/48), and *se*D, *sel*J, and *se*R (2.1%, 1/48, respectively).

Lastly, the distribution of the *S. aureus* in relation to their genotypes and the corresponding sources of isolation is displayed in Table 3.

### 2.3. Characterization of the Staphylococcal Chromosomal Cassette mec (SCCmec)

**Figure 2:**
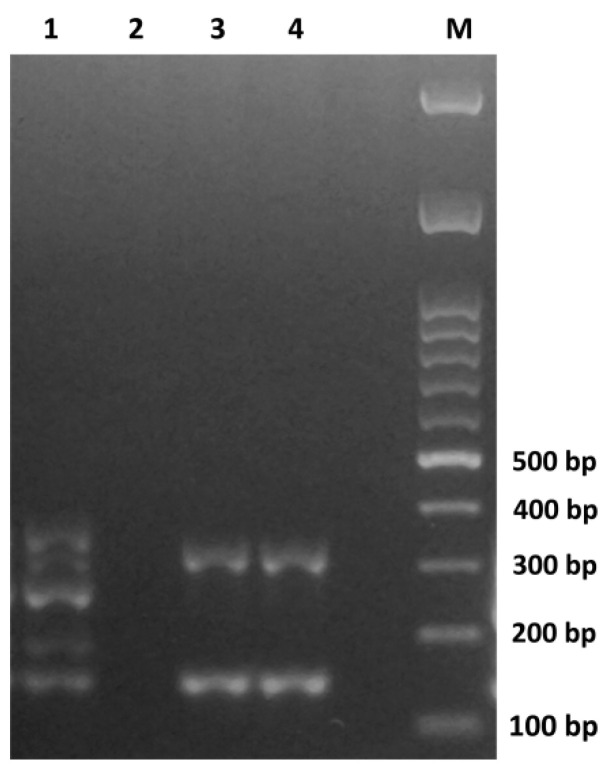
Characterization by multiplex PCR [30] of the staphylococcal chromosomal cassette *mec* (SCC*mec*) in *mec*A positive isolates from Algerian ready-to-eat foods. Lane 1: positive control (MRSA strain IMM1-T002 01-04 kindly provided by Prof. Karsten Becker, University Hospital Münster, Institute of Medical Microbiology, Münster, Germany); Lane 2: negative control using nuclease free water instead of DNA in PCR reaction mixture; Lane 3: *S. aureus* SA46; Lane 4: *S. aureus* SA17; M: DNA ladder (DM2300 ExcelBand™ 100 bp + 3K, Smobio Technology Inc., Taiwan).

The multiplex PCR used for the characterization of the staphylococcal chromosomal cassette *mec* (SCC*mec*) [30] showed that the two *mec*A positive isolates (Table 2) harbored the SCC*mec*-type IV as identified by the presence of a band of 342 bp (while the band of 162 bp indicates the presence of the *mec*A gene) (Figure 2).

### 2.4. Antimicrobial Resistance

The antimicrobial susceptibility test showed that 8 out of the 12 (66.7%) selected *S. aureus* strains (one for each ISR-type) isolated from ready-to-eat foods from Algeria were resistant to one antibiotic, i.e., benzylpenicillin, while three strains (25.0%) were resistant towards three to four antimicrobials (Table 4). Four resistance profiles were observed (Table 4). The highest prevalence of resistance was recorded for benzylpenicillin (10/12) (83.3%), while lower resistance was recorded towards tetracycline and kanamycin (2/12 strains, respectively) (16.7%, respectively) and towards ofloxacin, erythromycin, lincomycin, oxacillin, and cefoxitin (1/12 strains, respectively) (8.3%, respectively) (Table 4). One strain (1/12) (8.3%) (SA46) was considered MRSA, while two strains (2/12) (16.7%) (SA02, SA18) were multidrug-resistant.

## 3. Discussion

In the present study, we found *S. aureus* in Algerian ready-to-eat foods with a prevalence of 23.2%. Other studies analyzed the presence of this microorganism in ready-to-eat foods in African countries, but only fragmented and incomplete information about the enterotoxigenic *S. aureus* and MRSA isolated from African ready-to-eat foods is available so far. Similar to our findings, Chaalal et al. [31] reported an overall *S. aureus* prevalence of 23.8% in pastries and cooked dishes sampled from supermarkets and university cities in Western Algeria, and Titouche et al. [32], in the same country of the aforementioned study, found a prevalence of 14.46% in pastries collected from several market points (in Tizi Ouzou area), while no *S. aureus* contamination was found by these authors in the sampled sandwiches [32]. A prevalence of 33.26% was reported in ready-to-eat meat products collected in all the provinces of South Africa [33], while in Egypt (in Benha city), a prevalence of 50.8% was reported for ready-to-eat meat products sampled from restaurants and street vendors [34], and in Nigeria (in Port Harcourt Metropolis), 100% of the analyzed street ready-to-eat meals were reported to be contaminated by *S. aureus* [35].

As shown by these studies, the prevalence of *S. aureus* contamination in ready-to-eat foods may greatly vary and, as highlighted by some authors [32,36], such differences are related to several factors that include the source (e.g., street-vendors or shops) and type of samples (e.g., animal- or non-animal-derived foods), the sample size, the accuracy of the identification method (based on cultivation characteristics, biochemical tests, or molecular biology techniques), the manufacturing procedures (e.g., involving bactericidal temperatures or not), and the overall hygienic measures that are implemented during the preparation and handling of the foods. In particular, *S. aureus*, being a commensal bacterium present on the skin, the nose, and mucous membranes of animals and humans [28], may contaminate food especially when poor hygienic practices and conditions exist. It is noteworthy that, beyond the contamination that may originate from animals at the primary production stage, food handlers are recognized as the main source of food contamination with *S. aureus* [31,32,37], and Sezer et al. [38] found that the 79% of the food handlers employed in a catering establishment were carriers of *S. aureus*. Nevertheless, it should be taken into account that contamination may also happen and spread by cross-contamination among foods and/or surfaces in the environment surrounding the manufacturing and storage of the ready-to-eat foods, since *S. aureus* may survive on inanimate surfaces for prolonged times [39], especially in cases of improper or ineffective sanitizing procedures [40].

When contamination occurs, *S. aureus* may replicate, and its load may increase in food. *S. aureus* is mostly a coagulase positive bacterium, and according to the Interministerial Decree of the Algerian Republic Official Journal No 39 (published on 2 July 2017) establishing the microbiological criteria for food products, the limits of CPS in ready-to-eat foods are set to values equal to 10^2^ CFU/g (“m” value), below which the product quality is considered satisfactory, and 10^3^ CFU/g (“M” value), above which the product quality is considered unacceptable. In the ready-to-eat products sampled in this study, we found CPS loads, above the “M” value, in four of the five analyzed food categories (meat/fish-based foods, vegetable-based foods, pastries, and cereals), in which the highest CPS counts reached values higher than 4 and 5 Log CFU/g. Additionally, according to the Decree, samples in which CPS counts are superior or equal to 10^5^ CFU/g are considered as toxic. Therefore, these findings shed light on the quality and safety of the Algerian retail ready-to-eat foods.

Some other studies assessed CPS loads, even reported as *S. aureus* counts, in ready-to-eat foods in the African continent. Although Mahami et al. [41] found low contamination of cooked and smoked sausages, reporting *S. aureus* loads ranging from 1.85 to 2.15 Log CFU/g in samples purchased from a factory in Ghana (in Accra city) and less than 1 Log CFU/g in samples from a shopping mall, Oguttu et al. [42], analyzing ready-to-eat chicken sold in informal markets in South Africa (in Tshwane Metropolitan City), reported the presence of unsatisfactory quality samples with loads of *S. aureus* greater than 3 Log CFU/g, and an overall mean of 3.6 Log CFU/g. Similarly, Shiningeni et al. [43] found ready-to-eat beef and chicken meats of unsatisfactory quality, with loads of *S. aureus* equal or greater than 3 Log CFU/g, that were purchased from street vendors in Namibia (Windhoek city). These authors reported that, in relation to the vending sites, the mean counts in the ready-to-eat meat samples ranged from 0 to 3.46 Log CFU/g, and the highest load was 5.12 Log CFU/g [43].

Since the issue regarding *S. aureus* and corresponding loads in foods is related to the possible contamination of food by staphylococcal enterotoxins, that in very round figures may occur at hazardous levels if *S. aureus* load reaches around 5 Log CFU/g [40], a great importance was given in this study to the investigation of the enterotoxigenic potential of the *S. aureus* we isolated, and to the best of our knowledge this is the first study in Algeria in which the presence of a total of 27 *se* and *sel* genes was sought in foodborne *S. aureus*.

We found that the 48 *S. aureus* belonged to seven different SEg-types harboring the following combination of *se* and *sel* genes: (1) *sel*W, *sel*X; (2) *egc* (*se*G, *se*I, *se*M, *se*N, *se*O), *sel*W, *sel*X; (3) *se*A, *se*H, *se*K, *se*Q, *sel*W, *sel*X; (4) *se*B, *sel*W, *sel*X; (5) *se*D, *sel*J, *se*R, *sel*W, *sel*X; (6) *se*H, *sel*W, *sel*X, *sel*Y; and (7) *se*A, *egc* (*se*G, *se*I, *se*M, *se*N, *se*O), *sel*W, *sel*X. Previous studies detecting a high number of *se* and *sel* genes (17 to 27 genes) found 6 different genotypes in 53 *S. aureus* from raw milk [13], 11 different genotypes in 50 *S. aureus* from raw minced meat and sausages [44], and 120 enterotoxin gene patterns in a group comprising 568 *S. aureus* from humans, animals, foods, and the environment [45]. Our study corroborates these findings and indicates the heterogeneity of the enterotoxigenic potential that can be encountered in *S. aureus* isolates from ready-to-eat foods in Algeria.

In our study, we found that 100% (48/48) of the analyzed *S. aureus* harbored *sel*W and *sel*X. These are located in the chromosome and are reported as highly prevalent genes; indeed, they were detected in more than 92.0% and in more than 79.0% of the *S. aureus* analyzed in previous studies, respectively [46,47]. The recent identification of this and other new *se* and *sel* genes indicates that the pathogenic potential of *S. aureus* may be greater than previously thought [48]. Moreover, based on the findings of Aung et al. [46], it was suggested that *sel*W, and to a greater extent *sel*X, may play a universal role in the virulence of *S. aureus* [46].

The enterotoxin gene cluster (*egc*) was found in the 18.8% (9/48) of the *S. aureus* herein analyzed. This cluster, located in a variable genomic island (*v*Saβ) inserted in the chromosome [37], has been similarly reported with a prevalence of 16.2% [49] and of 26.4% [13] in *S. aureus* from various sources, including food. In our study, the presence of the *egc* was confirmed by the detection, in the same strains, of the *egc* encoded genes *se*G, *se*I, *se*M, *se*N, and *se*O. Various types of *egc* exist in *S. aureus* (*egc*1 to *egc*5) and, considering our findings, the strains herein analyzed may harbor *egc*1 [50] or the newly described *egc*5 [13]. It should be specified that these two *egc* types, beyond harboring the enterotoxin genes we detected (*se*G, *se*I, *se*M, *se*N, *se*O), might also include two pseudogenes (*ψent1-ψent2*) (*egc*1) or *sel*U2 (*egc*5) [13,50].

Since *sel*U2 results from a single adenine deletion in the *ψent1-ψent2* region (being just a 1 nucleotide frameshift of the *ψent1-ψent2* sequence that deletes the stop codon of pseudogene *ψent1*) [51], the design of primers able to specifically detect *ψent1-ψent2* or *sel*U2 is not achievable, making impracticable their easy and affordable detection by a PCR screening such as the one we performed.

The *se*H gene was detected in the *S. aureus* herein analyzed with a prevalence of 16.7% (8/48). This gene, located on a presumptive transposon [52], was reported with lower prevalence in some previous studies, being in the range of 4.05–4.8% in *S. aureus* from food and other sources [32,45], while a higher prevalence, more similar to our findings, and ranging between 24.6 and 28.0%, was reported by other authors analyzing *S. aureus* from raw milk, raw meat, and ready-to-eat foods [13,44,53].

The *egc*-encoded enterotoxins are reported with increasingly probability to have a role in staphylococcal foodborne poisoning (SFP) [54,55], and the *se*H encoded enterotoxin (SEH) is the first non-classical SE that has been reported to have caused SFP outbreaks [56,57], highlighting therefore the relevance of the detection of these genes in foodborne *S. aureus*.

When the classical *se* genes are considered, in our study we found *se*A, *se*B, and *se*D with a prevalence of 14.6% (7/48), 10.4% (5/48), and 2.1% (1/48), respectively. The presence of these genes, whose encoded enterotoxins are the most frequently reported causes of SFP outbreaks [58], is frequently investigated, and in African countries prevalences ranging between 6.7–90.0%, 0.0–18.3%, and 0.0–8.0% for each of them have been reported in *S. aureus* isolated from food products [32,59,60].

In our investigation, we also detected *sel*J, *se*K, *se*Q, *se*R, and *sel*Y. In general, scarce knowledge is available on the prevalence of these new *se* and *sel* genes in foodborne *S. aureus*. This study helps to improve this paucity of data, representing one of the few African reports available to date. Interestingly, we observed that some of the above-mentioned genes were found in association in the *S. aureus* herein analyzed, in particular, *sel*J and *se*R along with *se*D, and *se*K and *se*Q along with *se*A. The first association is carried on plasmids (pIB485-like), and the second is carried on prophages [37], and both of these associations are being found in *S. aureus* isolates from SFP outbreaks [61,62,63,64]. Considering that the emetic properties of the encoded SEs (SEK, SEQ, and SER) have been demonstrated [65,66], these new *se* genes may also have a role in the genesis of staphylococcal intoxication that to date is still not fully understood.

*tst* and *mec*A genes were found at low prevalence in the *S. aureus* analyzed in our study (2.1% (1/48) and 4.2% (2/48), respectively), and they were not in association in the same strains. These genes, being the genetic determinants of toxic shock syndrome toxin-1 (*tst*), which causes a range of systemic diseases, and methicillin resistance (*mec*A), which confers a general resistance to β-lactam antibiotics in MRSA, represent important virulence and antimicrobial resistance attributes that can worsen clinical conditions when *S. aureus* infections occur.

In general, a low prevalence of *tst* gene in foodborne *S. aureus* has also been reported in previous studies conducted in various parts of the world including African countries [31,67,68,69]. Although the association of *tst* and *mec*A genes has been found in *S. aureus* isolated especially from clinical samples [70,71], our findings resemble those of Chaalal et al. [31] that recently analyzed *S. aureus* from food products in Algeria detecting a low prevalence of *tst* gene (3.2%) that was found only in *mec*A-negative isolates.

Nevertheless, the presence of *mec*A has been reported in foodborne *S. aureus* isolates in Algeria, with a prevalence of 3.03 [72], 4.81 [32], and 16.9% [31], and, additionally, in the aforementioned studies, an overall low prevalence of MRSA in the analyzed foods has been reported, which is consistent with our findings [31,32,72]. The massive and inappropriate use of antimicrobials in veterinary and human medicine is considered the main cause for the emergence of antimicrobial-resistant strains [73]; however, the prevalence of MRSA in food is described to still be low and reported in the range of 1.6–6.4% in investigations also conducted in other countries [74].

Transmission of MRSA occurs between animals and humans but, notably, food contamination may serve as a vehicle to increase the dissemination of MRSA [73]. The community-acquired MRSA (CA-MRSA) but also the livestock-acquired MRSA (LA-MRSA) frequently carry the SCC*mec*-type IV [75,76], and they principally spread in human community settings and livestock populations. The detection of the SCC*mec*-type IV in our *S. aureus* isolates tentatively suggests their origin and, consistently with our findings, it was also the prevalent SCC*mec*-type found in the MRSA isolated from food in previous studies [31,77,78,79].

Interestingly, the ability of MRSA strains to act as a foodborne pathogens has been reported [80], and the first foodborne gastrointestinal illness outbreak caused by an enterotoxigenic MRSA strain has been described in 2002 [81]. However, to date, the actual MRSA involvement in SFP (cases or outbreaks) is not precisely elucidated, and a general underestimation is suggested [80]. Our *mec*A-SCCmec-type IV positive isolates harbored *se*H, *sel*W, *sel*X, and *sel*Y genes (SEg-type 6) suggesting their potential role as SFP agents, especially considering that the *se*H encoded enterotoxin (SEH) has been already reported to have caused SFP outbreaks [56,57], as mentioned above. Additionally, the association of *se*H and *mec*A gene is known and has been previously described [52]. Moreover, our results also corroborate those of previous studies that detected *se* and *sel* genes in MRSA isolated from food products [32,77,79,82].

To further characterize our *S. aureus* isolates, we employed ISR-PCR, previously used also by other authors as a practical tool for *S. aureus* genotyping [13,83]. Interestingly, unlike results previously reported by Chieffi et al. [13], we observed that ISR-PCR had a discriminatory power higher than SEg-typing since ISR-PCR enabled us to detect 12 ISR-types despite the seven SEg-types. On the other hand, we observed that *S. aureus* belonging to the same ISR-type harbored the same genes as detected by PCRs, corroborating the aforementioned study that reported that strains belonging to the same ISR-type showed the same SEg-type.

Therefore, ISR-PCR allowed us to select 12 representative genetically diverse *S. aureus* strains that, when tested for antimicrobial resistance, showed four patterns of resistance.

In particular, the antimicrobial susceptibility test allowed us to confirm one selected strain (1/12, 8.3%) as MRSA that is in agreement with the detection in the same strain of the *mec*A gene. Moreover, two selected strains (2/12, 16.7%) were classified as multidrug-resistant, being resistant to three (i.e., benzylpenicillin, kanamycin, and tetracycline) and four (i.e., ofloxacin, erythromycin, lincomycin, and tetracycline) antimicrobials belonging to different classes.

Multidrug-resistant *S. aureus* isolated from food products were detected also in previous studies conducted in Africa and in other parts of the world, whose reported prevalence ranged between 15.38–33.3% [31,32,84] and 10.4–57.5% [85,86], respectively. Such findings represent a threat to public health since the assortment of effective antimicrobials to treat *S. aureus* infections is reduced. Moreover, as for MRSA, these multidrug strains also harbored *se* and *sel* genes belonging to SEg-type 2 [*egc* (*se*G, *se*I, *se*M, *se*N, *se*O), *sel*W, *sel*X] and SEg-type 4 (*se*B, *sel*W, *sel*X), being therefore enterotoxigenic strains with the potential to cause SFP. Interestingly, the presence in food products of multidrug-resistant *S. aureus* strains carrying *se* and *sel* genes has also been reported by other authors [87].

The resistance to penicillin is reported with high prevalence; indeed, from 60% to more than 90% of foodborne *S. aureus* isolates showed penicillin resistance in many studies [31,32,85,86,88], which is consistent with the high prevalence of resistance observed also in our investigation (10/12, 83.3%).

We found that few isolates were resistant to tetracycline and kanamycin (2/12, 16.7%) and to ofloxacin, erythromycin, and lincomycin (1/12, 8.3%). Resistance to these antimicrobials has been reported also in previous studies with various prevalences of 17.31–54.1% [32,85,86,89], 10.2–32.6% [31,89], 0.0–12.4% [31,32], 5.77–52.1% [31,32,84,85,86,89], and 17.6% [31], respectively.

Some antimicrobials have an important role in the clinical practice, and among these, vancomycin is the drug of choice to treat serious infections caused by MRSA [90]. All our isolates were vancomycin-susceptible, which is consistent with other investigations that are reporting the absence of resistance to vancomycin in foodborne *S. aureus* [31,84,86,89].

## 4. Conclusions

The information on foodborne *S. aureus* in Africa is, to date, fragmented and still incomplete, calling for studies that can cover this lack of data from most of its countries. Herein, the detailed molecular characterization, in particular aimed to investigate the actual enterotoxigenic potential of the analyzed *S. aureus* isolates, along with the findings of MRSA and multidrug-resistant strains, highlight the pathogenicity as well as the heterogeneity of *S. aureus* population in Algerian ready-to-eat foods. Additionally, the finding of generally high CPS loads in the analyzed ready-to-eat foods, with respect to the microbiological criteria established by the Algerian legislation, draws attention to the quality and safety of such foods in Algeria.

Therefore, *S. aureus* contamination represents a current risk for consumers’ health, and ready-to-eat foods seem to still be far from the relevant Algerian standard, but we should point out that better hygienic practices during the manufacture of ready-to-eat foods may be an affordable strategy to successfully address these current issues.

## 5. Materials and Methods

### 5.1. Sampling

A total of 207 samples of ready-to-eat foods were randomly collected from hotels, restaurants, fast foods, and pizzerias in Algiers, capital of Algeria, during 2018 and 2019. Those samples included 55 meat-based foods, 72 vegetable-based foods, 43 pastries, 17 cereals, and 20 various foods (milk- and egg-based foods). The samples were transported on ice to the laboratory and analyzed immediately.

### 5.2. Isolation of Presumptive Staphylococcus aureus

The analysis was carried out using 25 g of homogenized food in 225 mL of pre-enrichment diluent tryptone-salt broth (Oxoid, Dardilly, France) using a Stomacher-type homogenizer. Further decimal dilutions were carried up to 10^−5^. Thereafter, the corresponding dilutions were plate-counted in accordance with the standard reference culture method recommended by the International Organization for Standardization [91] for the enumeration of coagulase-positive staphylococci, using Baird Parker with egg yolk emulsion (BPEY) incubated at 37 °C for 24–48 h. From each food sample processed, one presumptive *Staphylococcus aureus* colony was subcultured on BPEY (Oxoid, Dardilly, France) and purified by repeated streaking. The pure cultures were stored at −80 °C in brain heart infusion broth (BHI; Conda Pronadisa, Madrid, Spain), amended with 0.6% yeast extract (Biolife Italiana, Milano, Italy) added with 20% glycerol.

### 5.3. DNA Extraction

The presumptive *S. aureus* pure cultures were cultivated in BHI broth (Oxoid, Dardilly, France) amended with 0.6% yeast extract (Biolife Italiana, Milano, Italy) and incubated at 37 °C for 24 h. Five hundred microliters of each broth culture were centrifuged at 12,000 rpm for 90 s, and the resulting pellets were washed with 1 mL of sterilized distilled water. DNA was extracted with InstaGene Matrix (Bio-Rad, Hercules, CA, USA) following the manufacturer’s instructions. Three to five microliters of the resulting DNA solutions were used for the conventional polymerase chain reaction (PCR) protocols, while two microliters were used for the real time PCR protocols, as described below.

### 5.4. Identification of Staphylococcus aureus

The identification of *Staphylococcus aureus* isolates was carried out by a species-specific simplex polymerase chain reaction (PCR) targeting the *nuc* gene [92]. Briefly, each reaction mixture contained 3 µL of the extracted DNA, 0.4 µM of each primer, 2.5 mM of MgCl_2_ (Promega, Madison, WI, USA), 0.1 mM of each deoxynucleotide triphosphates (Promega, Madison, WI, USA), 0.8 U of GoTaq G2 hot start polymerase (Promega, Madison, WI, USA), 1X reaction buffer (Promega, Madison, WI, USA), and nucelase-free water to a final volume of 25 µL. Thermocycling conditions were the following: 94 °C for 2 min; 37 cycles of 94 °C for 1 min, 55 °C for 30 s, 72 °C for 1 min. 30 s.; final extension of 72 °C for 5 min. Amplicons were separated by electrophoresis in TAE buffer at 100V on agarose gel (1.5% *w*/*v*). *S. aureus* DSM20231^T^ was used as positive control.

### 5.5. Staphylococcal Enterotoxin Gene (SEg-) Typing and Real Time PCR of the Enterotoxin Gene Cluster (egc)

Conventional and real time PCR assays targeting the classical (*se*A to *se*E) and the newly described (*se*G to *sel*Z, *sel*27 and *sel*28) *se* and *sel* genes were carried out following the protocols described by Chieffi et al. [13], while the presence of the *egc* was assessed using the SYBR Green real-time PCR protocol described by Fusco et al. [11]. Amplicons obtained by conventional PCR were separated on agarose gel by electrophoresis in TAE buffer at 100V. *S. aureus* strains used as controls in the PCR assays are reported in Table S1 [13,50,93,94,95,96,97].

### 5.6. 16S-23S rDNA Intergenic Spacer Region PCR (ISR-PCR)

16S-23S rDNA intergenic spacer region PCR (ISR-PCR) was carried out using 5 µL of each *S. aureus* DNA following the protocol described by Chieffi et al. [13]. DNA of *S. aureus* DSM20231^T^ was also included in the PCR reaction as control reference strain. The resulting patterns were visualized as described by Fusco et al. [98], on agarose gel (1.7% *w*/*v*) electrophoresized in TAE buffer at 60 V for 6.5 h. Two patterns were considered different ISR-types if one or more DNA bands differed in size. The relevant analysis was carried out to construct an UPGMA (unweighted pair group method with arithmetic mean) dendrogram using the Bionumerics software version 5.1 (AppliedMaths, Sint Martens Latem, Belgium) [99], setting the Pearson correlation as a fingerprint similarity coefficient and choosing 1.0% position tolerance and 0.0% optimization as position tolerance settings.

### 5.7. Detection of tst and mecA Genes and Characterization of the Staphylococcal Chromosomal Cassette mec (SCCmec)

Five µL of *S. aureus* DNA were employed in the multiplex PCR protocol described by Oliveira and de Lencastre [30] for the detection of the *mec*A gene and the characterization of the SCC*mec* as well. The resulting amplicons were separated, loading 2 µL of the reaction mixture on agarose gel (2% *w*/*v*) and performing electrophoresis in TAE buffer at 100 V for 1 h 50 min. The *tst* gene was detected as described by Johnson et al. [100] with minor modifications in the reaction mixture that was prepared as follows: 3 µL of the extracted DNA, 0.4 µM of each primer, 2.5 mM of MgCl_2_ (Promega, Madison, WI, USA), 0.25 mM of each deoxynucleotide triphosphate (Promega, Madison, WI, USA), 1 U of GoTaq G2 hot start polymerase (Promega, Madison, WI, USA), 1X reaction buffer (Promega, Madison, WI, USA), and nucelase-free water to a final volume of 25 µL. Amplicons were separated by electrophoresis in TAE buffer at 100 V on agarose gel (1.3% *w*/*v*). *S. aureus* strains used as controls in the PCR assays are reported in Table S1 [13,50,93,94,95,96,97].

### 5.8. Antimicrobial Susceptibility Testing

Twelve *S. aureus* strains belonging to the different ISR-types were selected for testing (Table 4).

The pure cultures of each strain were cultured in Brain Heart Infusion (BHI) agar (Conda Pronadisa, Madrid, Spain) and incubated at 37 °C for 24 h. Suspensions of 0.5 McFarland were prepared from these cultures.

Antimicrobial susceptibility was performed by Vitek 2 (bioMérieux Inc., Durham, NC, USA) using AST-P631 cards. The cards were inoculated with the prepared suspensions and loaded into the Vitek 2 automated reader. Results were interpreted by the Vitek 2 Advanced Expert System software according to the reference criteria of CLSI and EUCAST [101,102]. *S*. *aureus* DSM 20231^T^ was used as control. Strains resistant to cefoxitin and oxacillin were considered MRSA, and strains resistant to ≥3 antimicrobials of different classes were considered multidrug-resistant. The following antimicrobial agents were included: benzylpenicillin, oxacillin, cefoxitin, gentamicin, kanamycin, tobramycin, ofloxacin, erythromycin, lincomycin, clindamycin, pristinamycin, linezolid, teicoplanin, vancomycin, tetracycline, fosfomycin, nitrofurantoin, fusidic acid, rifampicin, and co-trimoxazole.

## Figures and Tables

**Table 1:** Prevalence of *S. aureus* in ready-to-eat foods sampled in Algeria and related counts of coagulase positive staphylococci.

Food Category	Number of Samples	*S. aureus* Positive Samples (% of Positive Samples)	CPS Count ^a^	CPS Count Range ^b^
Meat/fish-based foods	55	21 (38.2%)	3.48 ± 0.86	1.40 ± 0.12–4.49 ± 0.26
Vegetable-based foods	72	16 (22.2%)	3.19 ± 1.03	1.00 ± 0.5–4.43 ± 0.15
Pastries	43	7 (16.3%)	3.57 ± 0.80	2.84 ± 0.11–5.11 ± 0.24
Cereals	17	3 (17.6%)	2.40 ± 1.10	2.08 ± 0.16–4.13 ± 0.14
Various foods (milk- and egg-based foods)	20	1 (5.0%)	1.70 ± 0.10	–
Total	207	48 (23.2%)	3.38 ± 0.94	1.00 ± 0.5–5.11 ± 0.24

^a^ Coagulase positive staphylococci count expressed as Log CFU/g ± standard deviation in *S. aureus* positive samples. The count is expressed as median ± standard deviation for the categories “meat/fish-based foods”, “vegetable-based foods”, “pastries”, and “cereals” where more than one *S. aureus* positive sample was found, and “total”. ^b^ Minimum and maximum counts of coagulase positive staphylococci found in *S. aureus* positive samples, expressed as Log CFU/g ± standard deviation.

**Table 2:** *S. aureus* isolated from Algerian ready-to-eat foods and corresponding genotypes, compared to *S. aureus* type strain DSM 20231.

Isolate	Origin	ISR-Type	SEg-Type ^a^	*tst*	*mec*A (SCC*mec* Type)
SA01	Potato in sauce ^b^	I	1	-	-
SA06	Chicken ^c^	I	1	-	-
SA11	Couscous with meat ^c^	I	1	-	-
SA13	Rice ^d^	I	1	-	-
SA23	Minced meat ^c^	I	1	-	-
SA33	Mashed potatoes ^b^	I	1	-	-
SA41	Salad ^b^	I	1	-	-
SA43	Rice ^d^	I	1	-	-
SA44	Salad ^b^	I	1	-	-
SA50	Salad ^b^	I	1	-	-
SA53	Minced meat ^c^	I	1	-	-
SA54	Beet salad ^b^	I	1	-	-
SA58	Meat ^c^	I	1	-	-
SA73	Salad ^b^	I	1	-	-
SA78	Lentil soup ^b^	I	1	-	-
SA83	Beans ^b^	I	1	-	-
SA84	Chicken ^c^	I	1	-	-
SA86	Sausages ^c^	I	1	-	-
SA87	Pastry ^e^	I	1	-	-
SA07	Meat ^c^	IV	1	-	-
SA22	Salad ^b^	IV	1	-	-
SA38	Spaghetti with tomato sauce ^b^	IV	1	-	-
SA39	Vegetable and meat sauce ^c^	IV	1	-	-
SA49	Potato ^b^	IV	1	-	-
SA10	Meat ^c^	VI	1	-	-
SA02	Meat ^c^	II	2	-	-
SA30	Meat ^c^	II	2	-	-
SA08	Meat ^c^	V	2	-	-
SA19	Pastry ^e^	IX	2	-	-
SA24	Lentil soup ^b^	IX	2	-	-
SA34	Meat ^c^	IX	2	-	-
SA37	Meat ^c^	IX	2	-	-
SA82	Sautéed beef with potato ^c^	XII	2	+	-
SA03	Salad ^b^	III	3	-	-
SA04	Meat ^c^	III	3	-	-
SA09	Pastry ^e^	III	3	-	-
SA29	Meat ^c^	III	3	-	-
SA31	Salad ^b^	III	3	-	-
SA72	Pastry ^e^	III	3	-	-
SA05	Pastry ^e^	VII	4	-	-
SA18	Pizza ^d^	VII	4	-	-
SA55	Pastry ^e^	VII	4	-	-
SA56	Pastry ^e^	VII	4	-	-
SA59	Russian salad ^b^	VII	4	-	-
SA20	Meat ^c^	VIII	5	-	-
SA17	Turkey pieces ^c^	X	6	-	+ (SCC*mec* IV)
SA46	Braised beef ^c^	X	6	-	+ (SCC*mec* IV)
SA51	Fermented milk ^f^	XI	7	-	-
DSM 20231^T^	Human pleural fluid	XIII	1	-	-

^a^ SEg-type: 1 (*sel*W, *sel*X); 2 [*egc* (*se*G, *se*I, *se*M, *se*N, *se*O), *sel*W, *sel*X]; 3 (*se*A, *se*H, *se*K, *se*Q, *sel*W, *sel*X); 4 (*se*B, *sel*W, *sel*X); 5 (*se*D, *sel*J, *se*R, *sel*W, *sel*X); 6 (*se*H, *sel*W, *sel*X, *sel*Y); 7 [*se*A, *egc* (*se*G, *se*I, *se*M, *se*N, *se*O), *sel*W, *sel*X]; ^b^ vegetable-based foods; ^c^ meat/fish-based foods; ^d^ cereals; ^e^ pastries; ^f^ various foods (milk- and egg-based foods).

**Table 3:** Distribution of *S. aureus* isolated from Algerian ready-to-eat foods based on their genotypes.

ISR-Type	SEg-Type ^a^	*tst*	*mec*A (SCC*mec* Type)	Origin and Number of *S. aureus* Isolates (% Out of the Total for Each Genotype)					Total (% of Total Isolates)
				Meat/Fish-Based Foods	Vegetable-Based Foods	Pastries	Cereals	Various Foods (Milk- and Egg-Based Foods)	
I	1	-	-	7 (36.8%)	9 (47.4%)	1 (5.3%)	2 (10.5%)		19 (39.6%)
IV	1	-	-	2 (40.0%)	3 (60.0%)				5 (10.4%)
VI	1	-	-	1 (100%)					1 (2.1%)
II	2	-	-	2 (100%)					2 (4.2%)
V	2	-	-	1 (100%)					1 (2.1%)
IX	2	-	-	2 (50.0%)	1 (25.0%)	1 (25.0%)			4 (8.3%)
XII	2	+	-	1 (100%)					1 (2.1%)
III	3	-	-	2 (33.3%)	2 (33.3%)	2 (33.3%)			6 (12.5%)
VII	4	-	-		1 (20.0%)	3 (60.0%)	1 (20.0%)		5 (10.4%)
VIII	5	-	-	1 (100%)					1 (2.1%)
X	6	-	+ (SCC*mec* IV)	2 (100%)					2 (4.2%)
XI	7	-	-					1 (100%)	1 (2.1%)
Total (% of total isolates)				21 (43.8%)	16 (33.3%)	7 (14.6%)	3 (6.2%)	1 (2.1%)	48 (100%)

^a^ SEg-type: 1 (*sel*W, *sel*X); 2 [*egc* (*se*G, *se*I, *se*M, *se*N, *se*O), *sel*W, *sel*X]; 3 (*se*A, *se*H, *se*K, *se*Q, *sel*W, *sel*X); 4 (*se*B, *sel*W, *sel*X); 5 (*se*D, *sel*J, *se*R, *sel*W, *sel*X); 6 (*se*H, *sel*W, *sel*X, *sel*Y); 7 [*se*A, *egc* (*se*G, *se*I, *se*M, *se*N, *se*O), *sel*W, *sel*X].

**Table 4:** Antimicrobial susceptibility of the selected *S. aureus* strains belonging to the different ISR-types, compared to *S. aureus* type strain DSM 20231.

Class	Antimicrobials	*S. aureus* Strains												
		SA01	SA02	SA04	SA07	SA08	SA10	SA18	SA20	SA24	SA46	SA51	SA82	DSM 20231^T^
β-Lactams	P	R	S	R	R	R	R	R	R	R	R	S	R	S
	OXA	S	S	S	S	S	S	S	S	S	R	S	S	S
	FOX	S	S	S	S	S	S	S	S	S	R	S	S	S
Aminoglycosides	GEN	S	S	S	S	S	S	S	S	S	S	S	S	S
	KA	S	S	S	S	S	S	R	S	S	R	S	S	S
	TOB	S	S	S	S	S	S	S	S	S	S	S	S	S
Quinolones	OF	S	R	S	S	S	S	S	S	S	S	S	S	S
Macrolides	ERY	S	R	S	S	S	S	S	S	S	S	S	I	S
Lincosamides	L	S	R	S	S	S	S	S	S	S	S	S	S	S
	CLI	S	S	S	S	S	S	S	S	S	S	S	S	S
Streptogramins	PRI	S	S	S	S	S	S	S	S	S	S	S	S	S
Oxazolidinones	LZ	S	S	S	S	S	S	S	S	S	S	S	S	S
Glycopeptides	TEI	S	S	S	S	S	S	S	S	S	S	S	S	S
	VAN	S	S	S	S	S	S	S	S	S	S	S	S	S
Tetracyclines	TE	S	R	S	S	S	S	R	S	S	S	S	S	S
Fosfomycins	FOS	S	S	S	S	S	S	S	S	S	S	S	S	S
Nitrofurans	NIT	S	S	S	S	S	S	S	S	S	S	S	S	S
Steroidal	FA	S	S	I	S	S	S	S	S	S	S	S	S	S
Ansamycins	RIF	S	S	S	S	S	S	S	S	S	S	S	S	S
Folate pathway inhibitors	COT	S	S	S	S	S	S	S	S	S	S	S	S	S
Resistance profile ^a^		P	OF, ERY, L, TE	P	P	P	P	P, KA, TE	P	P	P, OXA, FOX, KA	- ^b^	P	- ^b^

P: Benzylpenicillin, OXA: Oxacillin, FOX: Cefoxitin, GEN: Gentamicin, KA: Kanamycin, TOB: Tobramycin, OF: Ofloxacin, ERY: Erythromycin, L: Lincomycin, CLI: Clindamycin, PRI: Pristinamycin, LZ: Linezolid, TEI: Teicoplanin, VAN: Vancomycin, TE: Tetracyclin, FOS: Fosfomycin, NIT: Nitrofurantoin, FA: Fusidic acid, RIF: Rifampicin, COT: Co-trimoxazole; R: resistant; S: susceptible; I: intermediate; ^a^ antimicrobials to which the tested *S. aureus* strains are R; ^b^ sensitive to all tested antimicrobials.

## Supplementary Materials

The following are available online at https://www.mdpi.com/article/10.3390/toxins13120835/s1, Table S1: *S. aureus* strains used as controls in conventional and real time PCR assays in the present study.

## References

[1] Andjelković U., Šrajer Gajdošik M., Gašo-Sokač D., Martinović T., Josić D. (2017). Foodomics and Food Safety: Where We Are. Food Technol. Biotechnol..

[2] Jung Y., Jang H., Matthews K.R. (2014). Effect of the food production chain from farm practices to vegetable processing on outbreak incidence. Microb. Biotechnol..

[3] World Health Organization (2015). WHO Estimates of the Global Burden of Food Borne Diseases.

[4] Flynn K., Pérez Villarreal B., Barranco A., Belc N., Björnsdóttir B., Fusco V., Rainieri S., Smaradóttir S.E., Smeu I., Teixeira P. (2019). An introduction to current food safety needs. Trends Food Sci. Technol..

[5] McLinden T., Sargeant J.M., Thomas M.K., Papadopoulos A., Fazil A. (2014). Component costs of foodborne illness: A scoping review. BMC Public Health.

[6] Franz C.M.A.P., den Besten H.M.W., Bohnlein C., Gareis M., Zwietering M.H., Fusco V. (2018). Microbial food safety in the 21st century: Emerging challenges and foodborne pathogenic bacteria. Trends Food Sci Technol..

[7] Foster T.J. (2004). The *Staphylococcus aureus* “superbug”. J. Clin. Investig..

[8] Tong S.Y.C., Davis J.S., Eichenberger E., Holland T.L., Fowler V.G. (2015). *Staphylococcus aureus* infections: Epidemiology, pathophysiology, clinical manifestations, and management. Clin. Microbiol. Rev..

[9] Puah S.M., Chua K.H., Tan J.A. (2016). Virulence factors and antibiotic susceptibility of *Staphylococcus aureus* isolates in ready-to-eat foods: Detection of *S. aureus* contamination and a high prevalence of virulence genes. Int. J. Environ. Res. Public Health.

[10] Havelaar A.H., Kirk M.D., Torgerson P.R., Gibb H.J., Hald T., Lake R.J., Praet N., Bellinger D.C., de Silva N.R., Gargouri N. (2015). World Health Organization global estimates and regional comparisons of the burden of foodborne disease in 2010. PLoS Med..

[11] Fusco V., Quero G.M., Morea M., Blaiotta G., Visconti A. (2011). Rapid and reliable identification of *Staphylococcus aureus* harbouring the *enterotoxin gene cluster* (*egc*) and quantitative detection in raw milk by real time PCR. Int. J. Food Microbiol..

[12] Leyral G., Vierling E. (2007). Microbiologie et Toxicologie des Aliments: Hygiène et Sécurité Alimentaires.

[13] Chieffi D., Fanelli F., Cho G.-S., Schubert J., Blaiotta G., Franz C.M.A.P., Bania J., Fusco V. (2020). Novel insights into the enterotoxigenic potential and genomic background of *Staphylococcus aureus* isolated from raw milk. Food Microbiol..

[14] Cafini F., Le Thuy N.T., Román F., Prieto J., Dubrac S., Msadek T., Morikawa K. (2017). Methodology for the Study of Horizontal Gene Transfer in *Staphylococcus aureus*. J. Vis. Exp..

[15] Rajkovic A., Jovanovic J., Monteiro S., Decleer M., Andjelkovic M., Foubert A., Beloglazova N., Tsilla V., Sas B., Madder A. (2020). Detection of toxins involved in foodborne diseases caused by Gram-positive bacteria. Compr. Rev. Food Sci. Food Saf..

[16] Le Loir Y., Baron F., Gautier M. (2003). *Staphylococcus aureus* and food poisoning. Genet. Mol. Res..

[17] Gomes B.C., Franco B.D.G.M., De Martinis E.C.P. (2013). Microbiological food safety issues in Brazil: Bacterial pathogens. Foodborne Pathog. Dis..

[18] Chambers H.F., Deleo F.R. (2009). Waves of resistance: *Staphylococcus aureus* in the antibiotic era. Nat. Rev. Microbiol..

[19] Shlaes D.M., Projan S.J., Mayers D.L. (2009). Antimicrobial resistance versus the discovery and development of newantimicrobials. BT—Antimicrobial Drug Resistance: Mechanisms of Drug Resistance.

[20] Fishovitz J., Hermoso J.A., Chang M., Mobashery S. (2014). Penicillin-binding protein 2a of methicillin-resistant *Staphylococcus aureus*. IUBMB Life.

[21] Wang X., Li G., Xia X., Yang B., Xi M., Meng J. (2014). Antimicrobial susceptibility and molecular typing of methicillin-resistant *Staphylococcus aureus* in retail foods in Shaanxi, China. Foodborne Pathog. Dis..

[22] Islam M.A., Parveen S., Rahman M., Huq M., Nabi A., Khan Z.U.M., Ahmed N., Wagenaar J.A. (2019). Occurrence and Characterization of Methicillin Resistant *Staphylococcus aureus* in Processed Raw Foods and Ready-to-Eat Foods in an Urban Setting of a Developing Country. Front. Microbiol..

[23] Fusco V., Chieffi D., Fanelli F., Logrieco A.F., Cho G.-S., Kabisch J., Böhnlein C., Franz C.M.A.P. (2020). Microbial quality and safety of milk and milk products in the 21st century. Compr. Rev. Food Sci. Food Saf..

[24] Petinaki E., Spiliopoulou L. (2012). Methicillin-resistant *Staphylococcus aureus* among companion and food-chain animals: Impact of human contacts. Clin. Microbiol. Infect..

[25] Chiang Y.C., Liao W.W., Fan C.M., Pai W.Y., Chiou C.S., Tsen H.Y. (2008). PCR detection of Staphylococcal enterotoxins (SEs) N, O, P, Q, R, U, and survey of SE types in *Staphylococcus aureus* isolates from food-poisoning cases in Taiwan. Int. J. Food Microbiol..

[26] Chomvarin C., Chantarasuk Y., Srigulbutr S., Chareonsudjai S., Chaicumpar K. (2006). Enteropathogenic bacteria and enterotoxin-producing *Staphylococcus aureus* isolated from ready-to-eat foods in Khon Kaen, Thailand. Southeast Asian J. Trop. Med. Public Health.

[27] Oh S.K., Lee N., Cho Y.S., Shin D.B., Choi S.Y., Koo M. (2007). Occurrence of toxigenic *Staphylococcus aureus* in ready-to-eat food in Korea. J. Food Prot..

[28] Lozano C., Gharsa H., Ben Slama K., Zarazaga M., Torres C. (2016). *Staphylococcus aureus* in animals and food: Methicillin resistance, prevalence and population structure. A review in the African Continent. Microorganisms.

[29] Paudyal N., Anihouvi V., Hounhouigan J., Matsheka M.I., Sekwati-Monang B., Amoa-Awua W., Atter A., Ackah N.B., Mbugua S., Asagbra A. (2017). Prevalence of foodborne pathogens in food from selected African countries—A meta-analysis. Int. J. Food Microbiol..

[30] Oliveira D.C., de Lencastre H. (2002). Multiplex PCR strategy for rapid identification of structural types and variants of the *mec* element in methicillin-resistant *Staphylococcus aureus*. Antimicrob. Agents Chemother..

[31] Chaalal W., Chaalal N., Bourafa N., Kihal M., Diene S.M., Rolain J.-M. (2018). Characterization of *Staphylococcus aureus* Isolated from Food Products in Western Algeria. Foodborne Pathog. Dis..

[32] Titouche Y., Houali K., Ruiz-Ripa L., Vingadassalon N., Nia Y., Fatihi A., Cauquil A., Bouchez P., Bouhier L., Torres C. (2020). Enterotoxin genes and antimicrobial resistance in *Staphylococcus aureus* isolated from food products in Algeria. J. Appl. Microbiol..

[33] Madoroba E., Magwedere K., Chaora N.S., Matle I., Muchadeyi F., Mathole M.A., Pierneef R. (2021). Microbial communities of meat and meat products: An exploratory analysis of the product quality and safety at selected enterprises in South Africa. Microorganisms.

[34] Saad M.S., Fatin S.H., Fahim A.S., Marionette Z.N., Marwa Z.S. (2019). Prevalence of methicillin-resistant *Staphylococcus aureus* in some ready-to-eat meat products. Am. J. Biomed. Sci. Res..

[35] Ire S.F., Imuh V.T. (2016). Bacteriological quality evaluation and safety of randomly selected ready-to-eat foods sold in Port Harcourt city, Nigeria. J. Appl. Life Sci. Int..

[36] Abdeen E., Mousa W., Abdelsalam S., Heikal H., Shawish R., Nooruzzaman M., Soliman M., Batiha G., Hamad A., Abdeen A. (2021). Prevalence and Characterization of Coagulase Positive *Staphylococci* from Food Products and Human Specimens in Egypt. Antibiotics.

[37] Argudín M.Á., Mendoza M.C., Rodicio M.R. (2010). Food Poisoning and Staphylococcus aureus Enterotoxins. Toxins.

[38] Sezer Ç., Özgür Ç., Aksem A., Leyla V. (2015). Food handlers: A bridge in the journey of enterotoxigenic MRSA in food. J. Verbr. Lebensm..

[39] Kadariya J., Smith T.C., Thapaliya D. (2014). *Staphylococcus aureus* and staphylococcal food-borne disease: An ongoing challenge in public health. BioMed Res. Int..

[40] Prado Martin J.G., de Oliveira E., Silva G., da Fonseca C.R., Morales C.B., Souza Pamplona Silva C., Miquelluti D.L., Porto E. (2016). Efficiency of a cleaning protocol for the removal of enterotoxigenic *Staphylococcus aureus* strains in dairy plants. Int. J. Food Microbiol..

[41] Mahami T., Amafu-Dey H., Odonkor S. (2012). Microbial food safety risk: Cooked and smoked sausages as a potential source. Int. J. Biol. Pharm. Allied Sci..

[42] Oguttu J.W., McCrindle C.M.E., Makita K., Grace D. (2014). Investigation of the food value chain of ready-to-eat chicken and the associated risk for staphylococcal food poisoning in Tshwane Metropole, South Africa. Food Control.

[43] Shiningeni D., Chimwamurombe P., Shilangale R., Misihairabgwi J. (2019). Prevalence of pathogenic bacteria in street vended ready-to-eat meats in Windhoek, Namibia. Meat Sci..

[44] Bania J., Dabrowska A., Bystron J., Korzekwa K., Chrzanowska J., Molenda J. (2006). Distribution of newly described enterotoxin-like genes in Staphylococcus aureus from food. Int. J. Food Microbiol..

[45] Chao G., Bao G., Cao Y., Yan W., Wang Y., Zhang X., Zhou L., Wu Y. (2015). Prevalence and diversity of enterotoxin genes with genetic background of *Staphylococcus aureus* isolates from different origins in China. Int. J. Food Microbiol..

[46] Aung M.S., Urushibara N., Kawaguchiya M., Ito M., Habadera S., Kobayashi N. (2020). Prevalence and Genetic Diversity of Staphylococcal Enterotoxin (-Like) Genes sey, selw, selx, selz, sel26 and sel27 in Community-Acquired Methicillin-Resistant Staphylococcus aureus. Toxins.

[47] Wilson G.J., Tuffs S.W., Wee B.A., Seo K.S., Park N., Connelley T., Guinane C.M., Morrison W.I., Fitzgerald J.R. (2018). Bovine *Staphylococcus aureus* superantigens stimulate the entire T cell repertoire of cattle. Infect. Immun..

[48] Bianchi D., Gallina S., Bellio A., Chiesa F., Civera T., Decastelli L. (2014). Enterotoxin gene profiles of Staphylococcus aureus isolated from milk and dairy products in Italy. Lett. Appl. Microbiol..

[49] Song M., Shi C., Xu X., Shi X. (2016). Molecular typing and virulence gene profiles of enterotoxin gene cluster (egc)-positive *Staphylococcus aureus* isolates obtained from various food and clinical specimens. Foodborne Pathog. Dis..

[50] Jarraud S., Peyrat M.A., Lim A., Tristan A., Bes M., Mougel C., Etienne J., Vandenesch F., Bonneville M., Lina G. (2001). *egc*, a highly prevalent operon of enterotoxin gene, forms a putative nursery of superantigens in *Staphylococcus aureus*. J. Immunol..

[51] Collery M.M., Smyth C.J. (2007). Rapid differentiation of *Staphylococcus aureus* isolates harbouring *egc* loci with pseudogenes *ψent*1 and *ψent*2 and the *selu* or *selu_v_* gene using PCR-RFLP. J. Med. Microbiol..

[52] Noto M.J., Archer G.L. (2006). A subset of *Staphylococcus aureus* strains harboring staphylococcal cassette chromosome mec (SCCmec) type IV is deficient in CcrAB-mediated SCCmec excision. Antimicrob. Agents Chemother..

[53] Yang X., Yu S., Wu Q., Zhang J., Wu S., Rong D. (2018). Multilocus sequence typing and virulence-associated gene profile analysis of *Staphylococcus aureus* isolates from retail ready-to-eat food in China. Front. Microbiol..

[54] Johler S., Giannini P., Jermini M., Hummerjohann J., Baumgartner A., Stephan R. (2015). Further evidence for staphylococcal food poisoning outbreaks caused by *egc*-encoded enterotoxins. Toxins.

[55] Umeda K., Nakamura H., Yamamoto K., Nishina N., Yasufuku K., Hirai Y., Hirayama T., Goto K., Hase A., Ogasawara J. (2017). Molecular and epidemiological characterization of staphylococcal foodborne outbreak of *Staphylococcus aureus* harboring *seg*, *sei*, *sem*, *sen, seo*, and *selu* genes without production of classical enterotoxins. Int. J. Food Microbiol..

[56] Ikeda T., Tamate N., Yamaguchi K., Makino S. (2005). Mass outbreak of food poisoning disease caused by small amounts of staphylococcal enterotoxins A and H. Appl. Environ. Microbiol..

[57] Jørgensen H.J., Mathisen T., Lovseth A., Omoe K., Qvale K.S., Loncarevic S. (2005). An outbreak of staphylococcal food poisoning caused by enterotoxin H in mashed potato made with raw milk. FEMS Microbiol. Lett..

[58] Benkerroum N. (2017). Staphylococcal enterotoxins and enterotoxin-like toxins with special reference to dairy products: An overview. Crit. Rev. Food Sci. Nutr..

[59] Bissong M.E.A., Tahnteng B.F., Ateba C.N., Akoachere J.-F.T.K. (2020). Pathogenic Potential and Antimicrobial Resistance Profile of Staphylococcus aureus in Milk and Beef from the Northwest and Southwest Regions of Cameroon. BioMed Res. Int..

[60] Omwenga I., Aboge G.O., Mitema E.S., Obiero G., Ngaywa C., Ngwili N., Wamwere G., Wainaina M., Bett B. (2019). *Staphylococcus aureus* enterotoxin genes detected in milk from various livestock species in northern pastoral region of Kenya. Food Control.

[61] Chen Q., Xie S. (2019). Genotypes, enterotoxin gene profiles, and antimicrobial resistance of *Staphylococcus aureus* associated with foodborne outbreaks in Hangzhou, China. Toxins.

[62] Ciupescu L.-M., Auvray F., Nicorescu I.M., Meheut T., Ciupescu V., Lardeux A.-L., Tanasuica R., Hennekinne J.-A. (2018). Characterization of *Staphylococcus aureus* strains and evidence for the involvement of non-classical enterotoxin genes in food poisoning outbreaks. FEMS Microbiol. Lett..

[63] Denayer S., Delbrassinne L., Nia Y., Botteldoorn N. (2017). Food-Borne outbreak investigation and molecular typing: High diversity of *Staphylococcus aureus* strains and importance of toxin detection. Toxins.

[64] Sato’o Y., Omoe K., Naito I., Ono H.K., Nakane A., Sugai M., Yamagishi N., Hu D. (2014). Molecular epidemiology and identification of a *Staphylococcus aureus* clone causing food poisoning outbreaks in Japan. J. Clin. Microbiol..

[65] Omoe K., Hu D.-L., Ono H.K., Shimizu S., Takahashi-Omoe H., Nakane A., Uchiyama T., Shinagawa K., Imanishi K. (2013). Emetic potentials of newly identified staphylococcal enterotoxin-like toxins. Infect. Immun..

[66] Ono H.K., Omoe K., Imanishi K., Iwakabe Y., Hu D.L., Kato H., Saito N., Nakane A., Uchiyama T., Shinagawa K. (2008). Identification and characterization of two novel staphylococcal enterotoxins, types S and T. Infect. Immun..

[67] Aydin A., Sudagidan M., Muratoglu K. (2011). Prevalence of staphylococcal enterotoxins, toxin genes and genetic-relatedness of foodborne Staphylococcus aureus strains isolated in the Marmara Region of Turkey. Int. J. Food Microbiol..

[68] Jans C., Merz A., Johler S., Younan M., Tanner S.A., Kaindi D.W.M., Wangoh J., Bonfoh B., Meile L., Tasara T. (2017). East and West African milk products are reservoirs for human and livestock-associated *Staphylococcus aureus*. Food Microbiol..

[69] Wongboot W., Chomvarin C., Namwat W. (2015). Phenotypic and genotypic detection of enterotoxins, toxic shock syndrome toxin-1 and of methicillin resistance in *Staphylococcus aureus* isolated from retail ready-to-eat foods in Northeastern Thailand. Southeast Asian J. Trop. Med. Public Health.

[70] Durand G., Bes M., Meugnier H., Enright M.C., Forey F., Liassine N., Wenger A., Kikuchi K., Lina G., Vandenesch F. (2006). Detection of new methicillin-resistant *Staphylococcus aureus* clones containing the toxic shock syndrome toxin 1 gene responsible for hospital- and community-acquired infections in France. J. Clin. Microbiol..

[71] Sultan A.M., Nabiel Y. (2019). Association of *tsst*-1 and *pvl* with *mec*A genes among clinical *Staphylococcus aureus* isolates from a tertiary care hospital. J. Pure Appl. Microbiol..

[72] Achek R., Hotzel H., Cantekin Z., Nabi I., Hamdi T.M., Neubauer H., El-Adawy H. (2018). Emerging of antimicrobial resistance in staphylococci isolated from clinical and food samples in Algeria. BMC Res. Notes.

[73] Oniciuc E.-A., Nicolau A.I., Hernández M., Rodríguez-Lázaro D. (2017). Presence of methicillin-resistant *Staphylococcus aureus* in the food chain. Trends Food Sci. Technol..

[74] Crago B., Ferrato C., Drews S.J., Svenson L.W., Tyrrell G., Louie M. (2012). Prevalence of *Staphylococcus aureus* and methicillin-resistant *S. aureus* (MRSA) in food samples associated with foodborne illness in Alberta, Canada from 2007 to 2010. Food Microbiol..

[75] Mairi A., Touati A., Lavigne J.-P. (2020). Methicillin-Resistant *Staphylococcus aureus* ST80 Clone: A systematic review. Toxins.

[76] Monecke S., Slickers P., Gawlik D., Müller E., Reissig A., Ruppelt-Lorz A., Cortez de Jäckel S., Feßler A.T., Frank M., Hotzel H. (2018). Variability of SCCmec elements in livestock-associated CC398 MRSA. Vet. Microbiol..

[77] Aung K.T., Hsu L.Y., Koh T.H., Hapuarachchi H.C., Chau M.L., Gutiérrez R.A., Ng L.C., Hapuarachchi H.C. (2017). Prevalence of methicillin-resistant Staphylococcus aureus (MRSA) in retail food in Singapore. Antimicrob. Resist. Infect. Control.

[78] Liao F., Gu W., Yang Z., Mo Z., Fan L., Guo Y., Fu X., Xu W., Li C., Dai J. (2018). Molecular characteristics of *Staphylococcus aureus* isolates from food surveillance in southwest China. BMC Microbiol..

[79] Rodríguez-Lázaro D., Oniciuc E.-A., García P.G., Gallego D., Fernández-Natal I., Dominguez-Gil M., Eiros-Bouza J.M., Wagner M., Nicolau A.I., Hernández M. (2017). Detection and characterization of *Staphylococcus aureus* and methicillin-resistant *S. aureus* in foods confiscated in EU Borders. Front. Microbiol..

[80] Sergelidis D., Angelidis A. (2017). Methicillin-resistant *Staphylococcus aureus*: A controversial food-borne pathogen. Lett. Appl. Microbiol..

[81] Jones T.F., Kellum M.E., Porter S.S., Bell M., Schaffner W. (2002). An outbreak of community-acquired foodborne illness caused by methicillin-resistant *Staphylococcus aureus*. Emerg. Infect. Dis..

[82] Pu S., Wang F., Ge B. (2011). Characterization of toxin genes and antimicrobial susceptibility of *Staphylococcus aureus* isolates from Louisiana retail meats. Foodborne Pathog. Dis..

[83] Cremonesi P., Zottola T., Locatelli C., Pollera C., Castiglioni B., Scaccabarozzi L., Moroni P. (2013). Identification of virulence factors in 16S-23S rRNA intergenic spacer genotyped *Staphylococcus aureus* isolated from water buffaloes and small ruminants. J. Dairy Sci..

[84] Govender V., Madoroba E., Magwedere K., Fosgate G., Kuonza L. (2019). Prevalence and risk factors contributing to antibiotic-resistant *Staphylococcus aureus* isolates from poultry meat products in South Africa, 2015–2016. J. S. Afr. Vet. Assoc..

[85] Ge B., Mukherjee S., Hsu C.-H., Davis J.A., Tran T.T.T., Yang Q., Abbott J.W., Ayers S.L., Young S.R., Crarey E.T. (2017). MRSA and multidrug-resistant *Staphylococcus aureus* in U.S. retail meats, 2010–2011. Food Microbiol..

[86] Wang W., Baloch Z., Jiang T., Zhang C., Peng Z., Li F., Fanning S., Ma A., Xu J. (2017). Enterotoxigenicity and antimicrobial resistance of *Staphylococcus aureus* isolated from retail food in China. Front. Microbiol..

[87] Ma Y., Zhao Y., Tang J., Tang C., Chen J., Liu J. (2018). Antimicrobial susceptibility and presence of resistance & enterotoxins/enterotoxinlikes genes in *Staphylococcus aureus* from food. CyTA J. Food.

[88] Abdalrahman L.S., Stanley A., Wells H., Fakhr M.K. (2015). Isolation, Virulence, and Antimicrobial Resistance of Methicillin-Resistant Staphylococcus aureus (MRSA) and Methicillin Sensitive Staphylococcus aureus (MSSA) Strains from Oklahoma Retail Poultry Meats. Int. J. Environ. Res. Public Health.

[89] Kim Y.H., Kim H.S., Kim S., Kim M., Kwak H.S. (2020). Prevalence and characteristics of antimicrobial-resistant *Staphylococcus aureus* and methicillin-resistant *Staphylococcus aureus* from retail meat in Korea. Food Sci. Anim. Resour..

[90] Foster T.J. (2017). Antibiotic resistance in Staphylococcus aureus. Current status and future prospects. FEMS Microbiol. Rev..

[91] (1999). Microbiology of Food and Animal Feeding Stuffs—Horizontal Method for the Enumeration of Coagulase-Positive Staphylococci (Staphylococcus aureus and Other Species)—Part 1: Technique Using Baird-Parker Agar Medium.

[92] Brakstad O.G., Aasbakk K., Maeland J.A. (1992). Detection of *Staphylococcus aureus* by polymerase chain reaction amplification of the nuc gene. J. Clin. Microbiol..

[93] Blaiotta G., Ercolini D., Pennacchia C., Fusco V., Casaburi A., Pepe O., Villani F. (2004). PCR detection of staphylococcal enterotoxin genes in Staphylococcus spp. strains isolated from meat and dairy products. Evidence for new variants of seG and seI in S. aureus AB-8802. J. Appl. Microbiol..

[94] Sergeev N., Volokhov D., Chizhikov V., Rasooly A. (2004). Simultaneous analysis of multiple staphylococcal enterotoxin genes by an oligonucleotide microarray assay. J. Clin. Microbiol..

[95] Collery M.M., Smyth D.S., Tumilty J.J., Twohig J.M., Smyth C.J. (2009). Associations between enterotoxin gene cluster types *egc*1, *egc*2 and *egc*3, agr types, enterotoxin and enterotoxin-like gene profiles, and molecular typing characteristics of human nasal carriage and animal isolates of *Staphylococcus aureus*. J. Med. Microbiol..

[96] Schubert J., Podkowik M., Bystron J., Bania J. (2016). Production of staphylococcal enterotoxins in microbial broth and milk by *Staphylococcus aureus* strains harboring *se**h* gene. Int. J. Food Microbiol..

[97] Thomas D.Y., Jarraud S., Lemercier B., Cozon G., Echasserieau K., Etienne J., Gougeon M.-L., Lina G., Vandenesch F. (2006). Staphylococcal enterotoxin-like toxins U2 and V, two new staphylococcal superantigens arising from recombination within the enterotoxin gene cluster. Infect. Immun..

[98] Fusco V., Quero G.M., Stea G., Morea M., Visconti A. (2011). Novel PCR-based identification of *Weissella confusa* using an AFLP-derived marker. Int. J. Food Microbiol..

[99] Di Lena M., Quero G.M., Santovito E., Verran J., De Angelis M., Fusco V. (2015). A selective medium for isolation and accurate enumeration of *Lactobacillus casei*-group members in probiotic milks and dairy products. Int. Dairy J..

[100] Johnson W.M., Tyler S.D., Ewan E.P., Ashton F.E., Pollard D.R., Rozee K.R. (1991). Detection of genes for enterotoxins, exfoliative toxins, and toxic shock syndrome toxin 1 in *Staphylococcus aureus* by the polymerase chain reaction. J. Clin. Microbiol..

[101] CLSI (Clinical and Laboratory Standards Institute) (2012). Performance Standards for Antimicrobial Susceptibility Testing.

[102] EUCAST (The European Committee on Antimicrobial Susceptibility Testing) (2012). Breakpoint Tables for Interpretation of MICs and Zone Diameters, Version 2.0. http://www.eucast.org/clinical_breakpoints/.

