# Supplementary material for: Prevalence, Enterotoxigenic Potential and Antimicrobial Resistance of Staphylococcus aureus and Methicillin-Resistant Staphylococcus aureus (MRSA) Isolated from Algerian Ready to Eat Foods
Source: Toxins (Basel). 2021 Nov 24;13(12):835. doi: 10.3390/toxins13120835 (PMC8707561; doi:10.3390/toxins13120835)
Supplement: Supplementary file 1 [file toxins-13-00835-s001.zip › toxins-1421168-supplementary.pdf]

Supplementary Materials: Prevalence,Enterotoxigenic Potential and Antimicrobial Resistance of *Staphylococcus aureus* and Methicillin-Resistant *Staphylococcus aureus* (MRSA) Isolated from Algerian Ready to Eat Foods

Omar Amine Mekhloufi, Daniele Chieffi, Abdelhamid Hammoudi, Sid Ahmed Bensefia, Francesca Fanelli and Vincenzina Fusco

Table S1. *S. aureus* strains used as controls in conventional and real time PCR assays in the present study.

***S. aureus* strain**	**Reported gene content**	**Reference**
DSM 20231^{T}	*nuc*	[93]
ATCC 14458	*se*B, *se*K, *se*Q	[93,94]
ATCC 27664 (FRI326)	*se*E	[93]
ATCC 19095 (FRI137)	*se*C, *se*H, *se*L, *egc*2 (*se*G, *se*I, *se*M, *se*N, *se*O, *sel*U)	[93,95,96]
A900322	*se*P, *egc*1 (*se*G, *se*I, *se*M, *se*N, *se*O, *ψent*1, *ψent*2)	[50,93–96]
A900624	*egc*4 (*se*G, *se*N, *se*O, *sel*U2, *sel*V)	[95,97]
AB-8802	*egc*3 (*se*Gv, *se*Iv, *se*Mv, *se*Nv, *se*Ov, *sel*Uv)	[93,95]
NCTC 9393	*se*D, *selJ*, *egc*[a] (*se*G, *se*I, *se*M, *se*N, *se*O)	[93]
RIMD 31092	*se*B, *se*C, *egc*[a] (*se*G, *se*I, *se*M, *se*N, *se*O), *tst*	[93]
200P	*se*A, *sel*W, *sel*X	[13]
211P	*se*H, *sel*W, *sel*X	[13]
356P	*se*C, *se*L, *egc*5 (*se*G, *se*I, *se*M, *se*N, *se*O, *sel*U2) *sel*W, *sel*X	[13]
363P	*se*P, *egc*1 (*se*G, *se*I, *se*M, *se*N, *se*O, *ψent*1, *ψent*2), *sel*W, *sel*X	[13]
364P	*egc*5 (*se*G, *se*I, *se*M, *se*N, *se*O, *sel*U2), *sel*W, *sel*X, *sel*Y, *sel*27, *sel*28	[13]
372P	*se*A, *se*D, *selJ*, *se*R, *sel*W, *sel*X	[13]
IMM1-T002 01-04[b]	*mec*A	[13]

[a]Putatively *egc*1 based on restriction endonuclease analysis of the *se*M–*se*G PCR fragment [93].
[b]Kindly provided by Prof. Karsten Becker, University Hospital Münster, Institute of Medical Microbiology, Münster, Germany.

References

13. Chieffi, D.; Fanelli, F.; Cho, G.-S.; Schubert, J.; Blaiotta, G.; Franz, C.M.A.P.; Bania, J.; Fusco, V. Novel insights into the enterotoxigenic potential and genomic background of *Staphylococcus aureus* isolated from raw milk. *Food Microbiol.* **2020**, *90*, 103482, doi:10.1016/j.fm.2020.103482.
50. Jarraud, S.; Peyrat, M.A.; Lim, A.; Tristan, A.; Bes, M.; Mougel, C.; Etienne, J.; Vandenesch, F.; Bonneville, M.; Lina, G. *egc*, a highly prevalent operon of enterotoxin gene, forms a putative nursery of superantigens in *Staphylococcus aureus*. *J. Immunol.* **2001**, *166*, 669–677, doi:10.4049/jimmunol.166.1.669.
93. Blaiotta, G.; Ercolini, D.; Pennacchia, C.; Fusco, V.; Casaburi, A.; Pepe, O.; Villani, F. PCR detection of staphylococcal enterotoxin genes in *Staphylococcus* spp. strains isolated from meat and dairy products. Evidence for new variants of *se*G and *se*I in *S. aureus* AB-8802. *J. Appl. Microbiol.* **2004**, *97*, 719–730, doi:10.1111/j.1365-2672.2004.02349.x.

94. Sergeev, N.; Volokhov, D.; Chizhikov, V.; Rasooly, A. Simultaneous analysis of multiple staphylococcal enterotoxin genes by an oligonucleotide microarray assay. *J. Clin. Microbiol.* **2004,** *42*, 2134–2143, doi:10.1128/jcm.42.5.2134-2143.2004.
95. Collery, M.M.; Smyth, D.S.; Tumilty, J.J.; Twohig, J.M.; Smyth, C.J. Associations between enterotoxin gene cluster types *egc*1, *egc*2 and *egc*3, agr types, enterotoxin and enterotoxin-like gene profiles, and molecular typing characteristics of human nasal carriage and animal isolates of *Staphylococcus aureus*. *J. Med. Microbiol.* **2009,** *58*, 13–25, doi:10.1099/jmm.0.005215-0.
96. Schubert, J.; Podkowik, M.; Bystron, J.; Bania, J. Production of staphylococcal enterotoxins in microbial broth and milk by *Staphylococcus aureus* strains harboring *seh* gene. *Int. J. Food Microbiol.* **2016**, *235*, 36–45, doi:10.1016/j.ijfoodmicro.2016.06.043.
97. Thomas, D.Y.; Jarraud, S.; Lemercier, B.; Cozon, G.; Echasserieau, K.; Etienne, J.; Gougeon, M.-L.; Lina, G.; Vandenesch, F. Staphylococcal enterotoxin-like toxins U2 and V, two new staphylococcal superantigens arising from recombination within the enterotoxin gene cluster. *Infect. Immun.* **2006,** *74*, 4724–4734, doi:10.1128/IAI.00132-06.
